# Treatment of Melasma Targeting Dermal–Epidermal Interactions Utilizing High‐Intensity, High‐Frequency Parallel Ultrasound Beam in Asian Skin

**DOI:** 10.1111/jocd.70820

**Published:** 2026-03-25

**Authors:** Kentaro Oku

**Affiliations:** ^1^ Hills Grace Clinic Yokohama Japan

**Keywords:** high frequency, melasma dermal–epidermal interactions senescent fibroblasts skin quality improvement thermal‐thread technique high‐intensity, parallel ultrasound beam Asian skin

## Abstract

**Background:**

Melasma is a multifactorial disorder, and while treatments aimed at suppressing melanin production and removing excess melanin have demonstrated some efficacy, no definitive therapy has yet been established. Dermal aging is widely recognized as a contributing factor in melasma, and previous studies have demonstrated that the presence of senescent fibroblasts reduces the efficacy of melasma treatments. Therefore, treatment strategies focused on reactivating fibroblast activity are anticipated to be effective against melasma.

**Objective:**

The objective of this study is to investigate the safety and efficacy of the Thermal‐Thread Technique, which utilizes high‐intensity, high‐frequency parallel ultrasound beams for the treatment of melasma among Asian subjects.

**Study Design/Methods:**

Patients diagnosed with melasma, regardless of disease type, duration, or Fitzpatrick skin type, underwent a single treatment session covering the entire face utilizing a high‐intensity, high‐frequency parallel ultrasound beam with Thermal‐Thread Technique. High‐resolution skin images were captured using a skin analyzer before treatment and six months after the treatment. Two independent evaluators assessed these images using the modified Melasma Area and Severity Index (mMASI) scoring system to objectively evaluate treatment efficacy. Statistical analyses of the mMASI scores were performed using paired *t*‐tests. All potential side effects were carefully monitored both during and after the procedure.

**Results:**

All patients (*n* = 20, female, mean age: 50.5 ± 5.7) completed the study. The distribution of Fitzpatrick skin types among participants was as follows: type II (*n* = 7), type III (*n* = 11), and type IV (*n* = 2). The mean mMASI score significantly decreased from 4.63 ± 1.66 at baseline to 1.69 ± 0.90 six months post‐treatment (*p* = 1.53e‐9; *p* < 0.001). No statistically significant difference was observed among FST groups (F(2,17) = 1.68, *p* = 0.216). No side effects were observed or reported during or after the treatment period.

**Conclusion:**

The improvement in mMASI scores observed with the Thermal‐Thread Technique, utilizing a high‐intensity, high‐frequency parallel ultrasound beam, demonstrates its potential as an effective treatment for melasma. Further research is necessary to evaluate its efficacy in more severe cases, extend the observational period, and investigate the potential benefits associated with multiple treatment sessions.

## 1 Introduction

Melasma is a common yet challenging dermatological condition characterized by hyperpigmented lesions predominantly affecting facial regions. It is a chronic and recurrent disorder frequently observed in sun‐exposed areas, notably the cheeks, forehead, upper lip, and nose. Clinically, melasma presents as symmetric, irregularly shaped patches exhibiting varying shades of brown and gray, significantly contributing to cosmetic concerns and adversely impacting patients' quality of life due to associated psychosocial distress. Histopathologically, melasma is characterized by increased melanin deposition within the epidermis and/or dermis, often accompanied by structural alterations in dermal and epidermal components. Pathophysiologically, melasma involves complex interactions among multiple contributing factors, including genetic predisposition, hormonal influences (such as pregnancy and oral contraceptive use), ultraviolet radiation exposure, and oxidative stress [1]. Recent investigations have further elucidated the critical role of dermal components, particularly fibroblasts and extracellular matrix proteins [2, 3]. In particular, it has been reported that the presence of senescent collagen fibers correlates with reduced therapeutic efficacy in melasma treatment [4]. Increasing evidence suggests that interactions between the dermis and epidermis significantly influence melanogenesis and melanin transfer. Additionally, chronic ultraviolet radiation exposure contributes to cumulative dermal damage, promoting the emergence of senescent fibroblasts. These fibroblasts secrete various pro‐inflammatory cytokines and growth factors, collectively known as the senescence‐associated secretory phenotype (SASP), exacerbating the condition by sustaining chronic inflammation and disrupting dermal–epidermal homeostasis [5].

Given the complexity and multifactorial nature of melasma, achieving sustained therapeutic efficacy remains challenging. Standard treatments primarily focus on reducing pigmentation by inhibiting melanin synthesis or transfer but frequently encounter limitations such as recurrence, insufficient resolution, and therapeutic resistance. Consequently, therapeutic strategies specifically targeting dermal–epidermal interactions and senescent fibroblast populations hold significant potential for enhancing treatment efficacy and durability. The Thermal‐Thread Technique, using high‐intensity, high‐frequency parallel ultrasound beams, selectively delivers thermal energy to dermal layers, aiming to modulate fibroblast activity, mitigate dermal senescence, and restore the activity of dermal–epidermal interactions, thereby potentially improving melasma management.

## 2 Methods

This prospective, uncontrolled clinical trial was conducted from November 2023 to November 2024 in accordance with the ethical principles of the Declaration of Helsinki. Written informed consent was obtained from all participants before enrollment. The study protocol was reviewed and approved by the Shiba Palace Clinic Institutional Review Board (Approval Number; 162120_rn‐42 485).

Twenty female subjects aged 40–65 years with a clinical diagnosis of melasma were prospectively enrolled in this uncontrolled clinical trial. Participants were included irrespective of melasma severity, duration of disease, or Fitzpatrick skin type. Comprehensive dermatological assessments, including Wood's lamp examination, were performed to confirm the diagnosis of melasma and determine study eligibility. The diagnosis was based on clinical features including pigmentation color, bilateral symmetry, and involvement of typical facial regions.

Exclusion criteria included active skin infections, any dermatological conditions potentially interfering with wound healing, therapeutic interventions (including ablative and non‐ablative procedures, botulinum toxin injections, or dermal filler treatments) performed within the previous 6 months, and topical, systemic (oral), or device‐based therapies specifically targeting melasma administered within the same 6‐month period preceding enrollment.

The study protocol consisted of two structured visits. The initial baseline visit encompassed a detailed clinical evaluation, photographic documentation, provision of detailed treatment information to participants, and acquisition of written informed consent, followed by a single treatment session using a high‐intensity, high‐frequency parallel ultrasound beam device, Sofwave (Sofwave Medical Ltd.). Following treatment, all subjects were instructed to apply sunscreen daily, irrespective of outdoor activity, and to reapply during daylight hours. In addition, subjects were instructed to use only a ceramide‐containing moisturizer twice daily and were prohibited from using depigmenting agents or anti‐aging formulations. The second visit took place 24 weeks post‐treatment, during which outcomes were thoroughly evaluated. Clinical photographs were captured using the high‐resolution skin analyzer system Re‐Beau 2 (JMEC Co. Ltd.). The severity of melasma was objectively assessed by two independent board‐certified dermatologists who were not involved in the study utilizing the modified Melasma Area and Severity Index (mMASI). Participant satisfaction was evaluated using a five‐point Likert scale: Very satisfied, Satisfied, Neutral, Dissatisfied, Very dissatisfied.

### 2.1 Intervention

Treatment preparation involved meticulous cleansing of the facial skin to completely remove makeup and skincare products. Subsequently, a topical anesthetic cream containing 10.56% lidocaine was uniformly applied for 30 min before treatment, ensuring patient comfort during the procedure. The ultrasound treatment was performed using the manufacturer's handpiece equipped with seven transducers arranged in parallel. Each transducer has a rectangular aperture measuring 1 mm × 5 mm, and a single shot generates seven parallel, cylindrical‐shaped thermal zones within the dermis. The intended focal depth of the thermal effect is approximately 1.5 mm. The handpiece incorporates contact cooling to protect the epidermis, and a post‐cooling phase is automatically applied after each shot. For the present protocol, each shot was delivered over 5.0 s, followed immediately by 1.0 s of post‐cooling.

Ultrasound energy settings were standardized to 3.2 J/shot for the submental, cheek, and temple regions and to 3.0 J/shot for the forehead, irrespective of subjects' skin type or melasma subtype.

**FIGURE 1:**
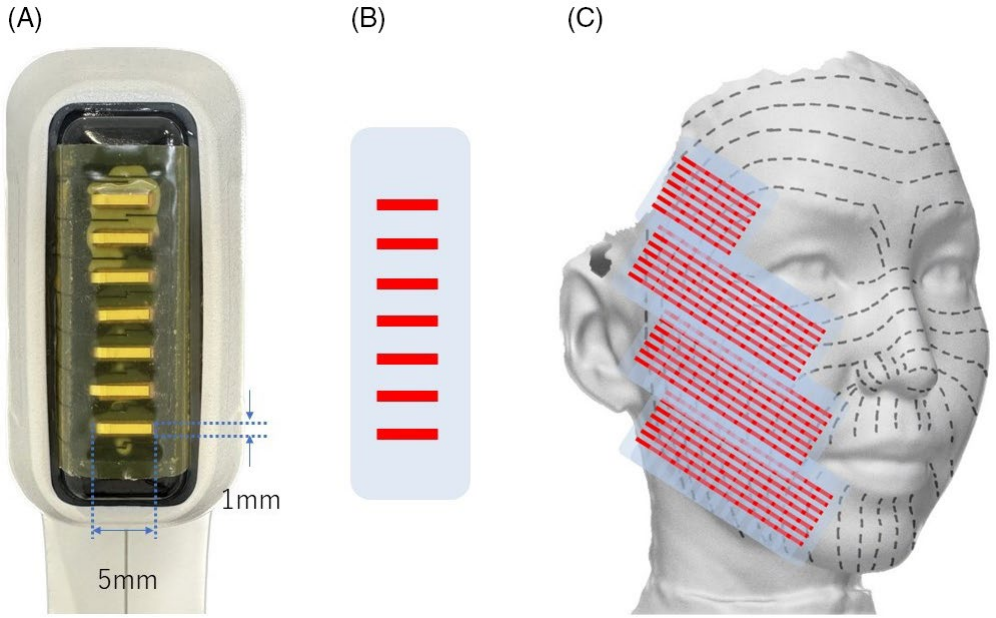
Application of shots using the Thermal‐Thread Technique on the right side. (A) Frontal view of the Sofwave transducer, illustrating seven vertically aligned rectangular transducers, each measuring 1 mm × 5 mm. (B) Schematic representation of the generated thermal zones. (C) Correct alignment and combination of shots without gaps, positioned perpendicularly to the relaxed skin tension lines (indicated by dashed lines).

The Thermal‐Thread Technique specifically refers to an ultrasound‐based thermal energy delivery method wherein the energy is applied perpendicular to the relaxed skin tension lines (RSTL) [6], as illustrated in Figure 1. Each irradiation lasted 5 s, followed immediately by a 1‐s post‐cooling phase, after which the handpiece was shifted parallel by 5 mm to the next adjacent treatment area within a 1‐s interval. Precise alignment of the transducer was critically maintained, ensuring perpendicular contact with the skin surface and consistent pressure throughout the treatment session. Any lateral deviation during parallel movement could disrupt thermal coupling, potentially compromising treatment effectiveness. This precise sequence was systematically repeated to ensure consistent thermal energy delivery and comprehensive coverage.

Pain experienced during the procedure was evaluated using a validated 5‐point Likert scale (1: no pain, 2: mild pain, 3: moderate pain, 4: severe pain, and 5: very severe pain). Additionally, any side effects or complications related to the procedure were meticulously documented by the dermatologist throughout both the treatment and the follow‐up periods.

Statistical analyses for evaluating the changes in mMASI scores were performed using a one‐sample *t*‐test. The mean of the mMASI scores assigned by two independent evaluators was used for the analysis. Statistical significance was set at the 5% level (*p* < 0.05 was considered statistically significant). Continuous variables were expressed as mean ± standard deviation. In addition, a one‐way analysis of variance ANOVA was performed to evaluate differences in the percentage improvement in mMASI among Fitzpatrick skin types with the percentage improvement for each subject used as the dependent variable and Fitzpatrick skin type used as the factor.

## 3 Results

**FIGURE 2:**
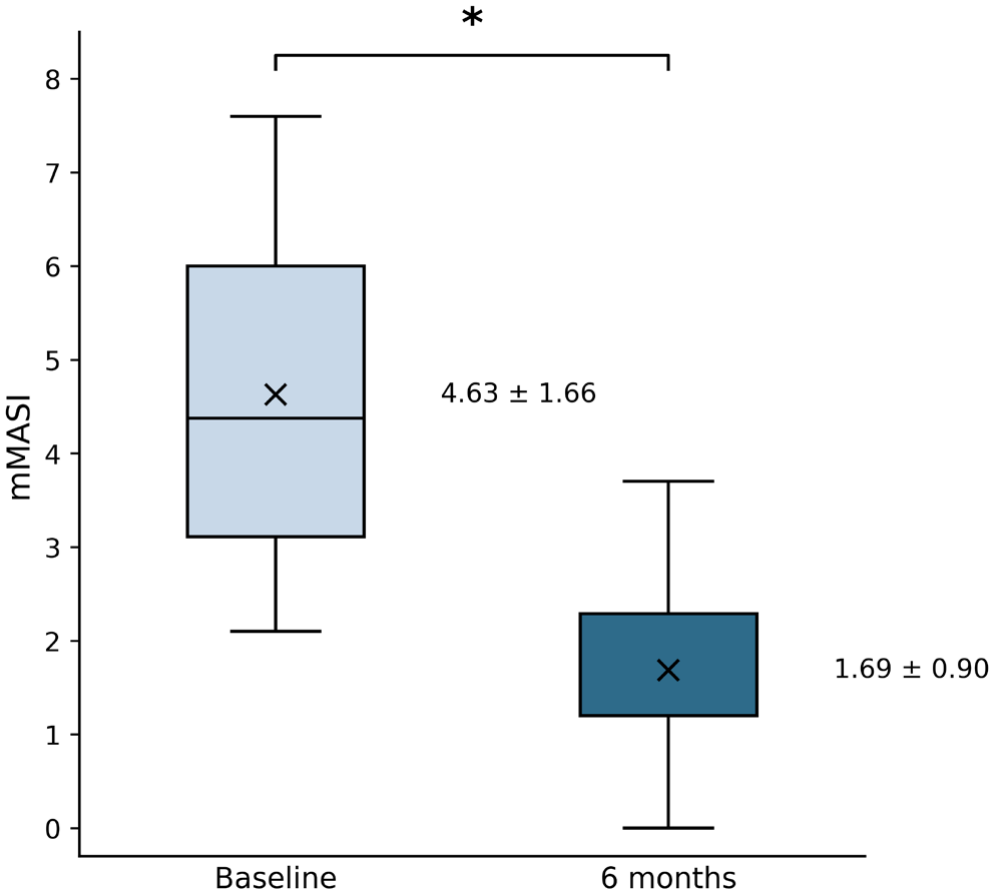
Boxplot of mean mMASI scores at baseline and 6 months post‐treatment. * Indicates a statistically significant decrease compared to the baseline.

All twenty patients completed the study protocol. The mean age of the participants was 50.5 ± 5.7 years (range 40–61). The distribution of Fitzpatrick skin types among subjects was as follows: type II (*n* = 7), type III (*n* = 11), and type IV (*n* = 2). At baseline, the mean mMASI score was 4.63 ± 1.66. Six months post‐treatment, the mean mMASI score significantly decreased to 1.69 ± 0.90 (*p* = 1.53e‐9; *p* < 0.001), corresponding to an average improvement rate of 64% (Figure 2). The percentage improvement in mMASI score, stratified by Fitzpatrick skin type, was as follows: FST II, 59.9%; FST III, 68.9%; and FST IV, 52.7%.

In subjects with Fitzpatrick skin type II (*n* = 7), the mean mMASI score decreased from 4.86 ± 1.25 at baseline to 1.99 ± 0.93 at 6 months post‐treatment, representing a statistically significant reduction (paired *t*‐test, *p* < 0.001). Similarly, in subjects with Fitzpatrick skin type III (*n* = 11), the mean mMASI score decreased from 4.39 ± 1.98 at baseline to 1.35 ± 0.79 at 6 months post‐treatment (paired *t*‐test, *p* < 0.001) (Table 1). To explore whether treatment response differed by phototype, one‐way ANOVA was performed on percentage mMASI improvement across Fitzpatrick skin types II–IV. The mean improvement rates were 59.9% (FST II, *n* = 7), 68.5% (FST III, *n* = 11), and 52.7% (FST IV, *n* = 2), with no statistically significant difference among groups (F(2,17) = 1.68, *p* = 0.22). Because the FST IV subgroup was small (*n* = 2), this subgroup analysis should be interpreted as exploratory.

**TABLE 1:** Baseline characteristics and mMASI scores stratified by fitzpatrick skin type (FST). individual subject ID, age, and modified melasma area and severity index (mMASI) scores at baseline (Pre‐mMASI) and at 6 months post‐treatment (Post‐mMASI) are shown for FST II, III, and IV. group summary values are presented as mean ± standard deviation.

FST	Subject ID	Age	Pre‐mMASI	Post‐mMASI
II	2	47	2.8	1.2
	8	40	4	1.2
	10	46	6	2.55
	12	42	4.05	1.2
	13	49	5.8	3.7
	16	51	5.4	2.25
	17	55	6	1.8
	Mean ± SD	47.14 ± 4.76	4.86 ± 1.16	1.99 ± 0.86
III	1	58	4.6	1.3
	3	41	2.1	0.9
	4	50	2.4	1.2
	7	55	4.05	1.2
	9	47	5.7	1.2
	11	49	3	1.2
	14	51	2.45	0
	15	50	3.15	1.2
	18	51	7.6	3.05
	19	55	6	2.4
	20	60	7.2	1.2
	Mean ± SD	51.55 ± 5.05	4.39 ± 1.89	1.35 ± 0.75
IV	5	61	6.15	3.15
	6	52	4.15	1.8
	Mean ± SD	56.50 ± 4.50	5.15 ± 1.00	2.48 ± 0.67

**FIGURE 3:**
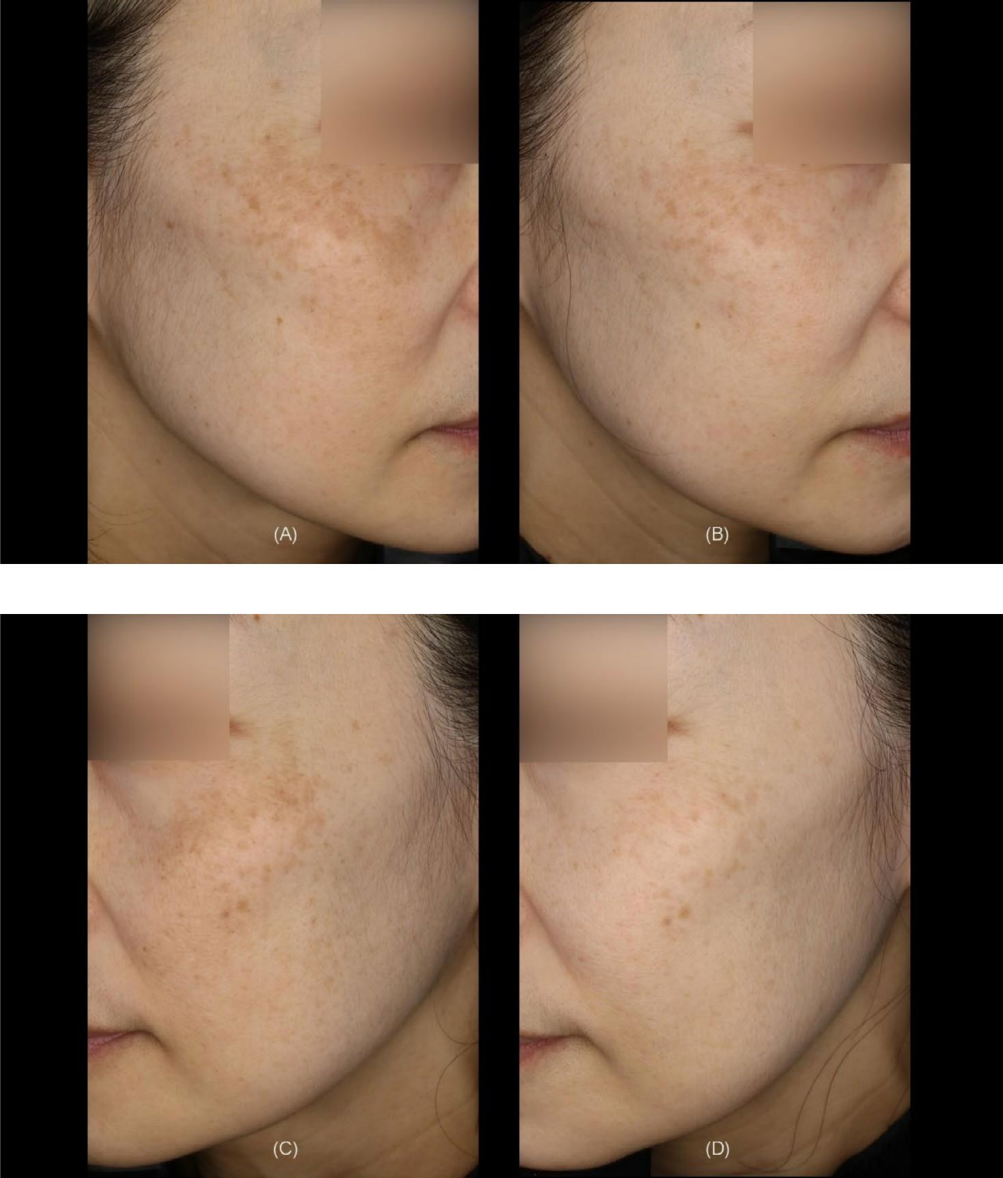
A 52‐year‐old female with Fitzpatrick Skin Type II. (A) Right side view at baseline. (B) Right side view at 6 months after the treatment. (C) Left side view at baseline. (D) Left side view at 6 months after the treatment.

**FIGURE 4:**
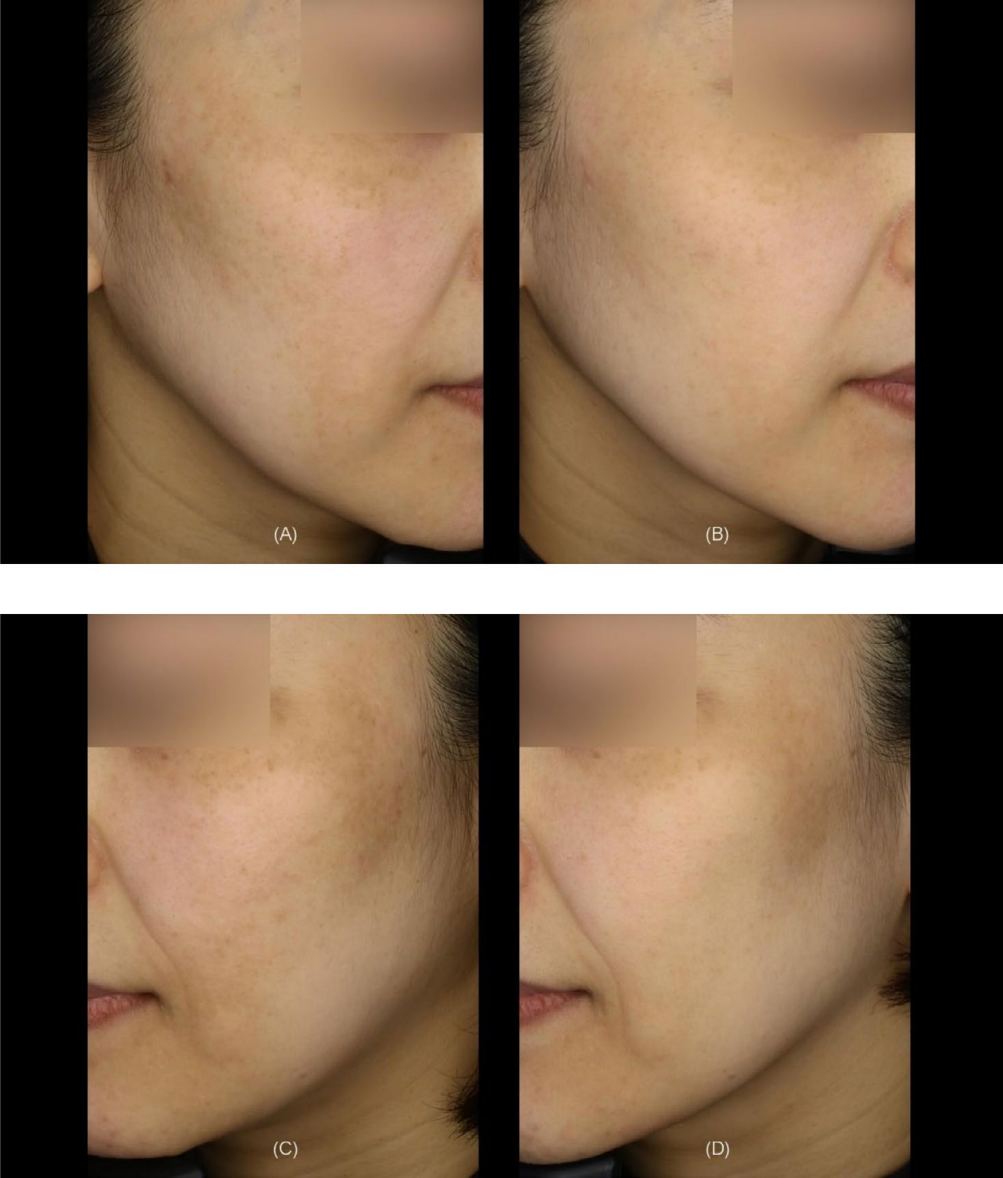
A 47‐year‐old female with Fitzpatrick Skin Type III. (A) Right side view at baseline. (B) Right side view at 6 months after the treatment. (C) Left side view at baseline. (D) Left side view at 6 months after the treatment.

**FIGURE 5:**
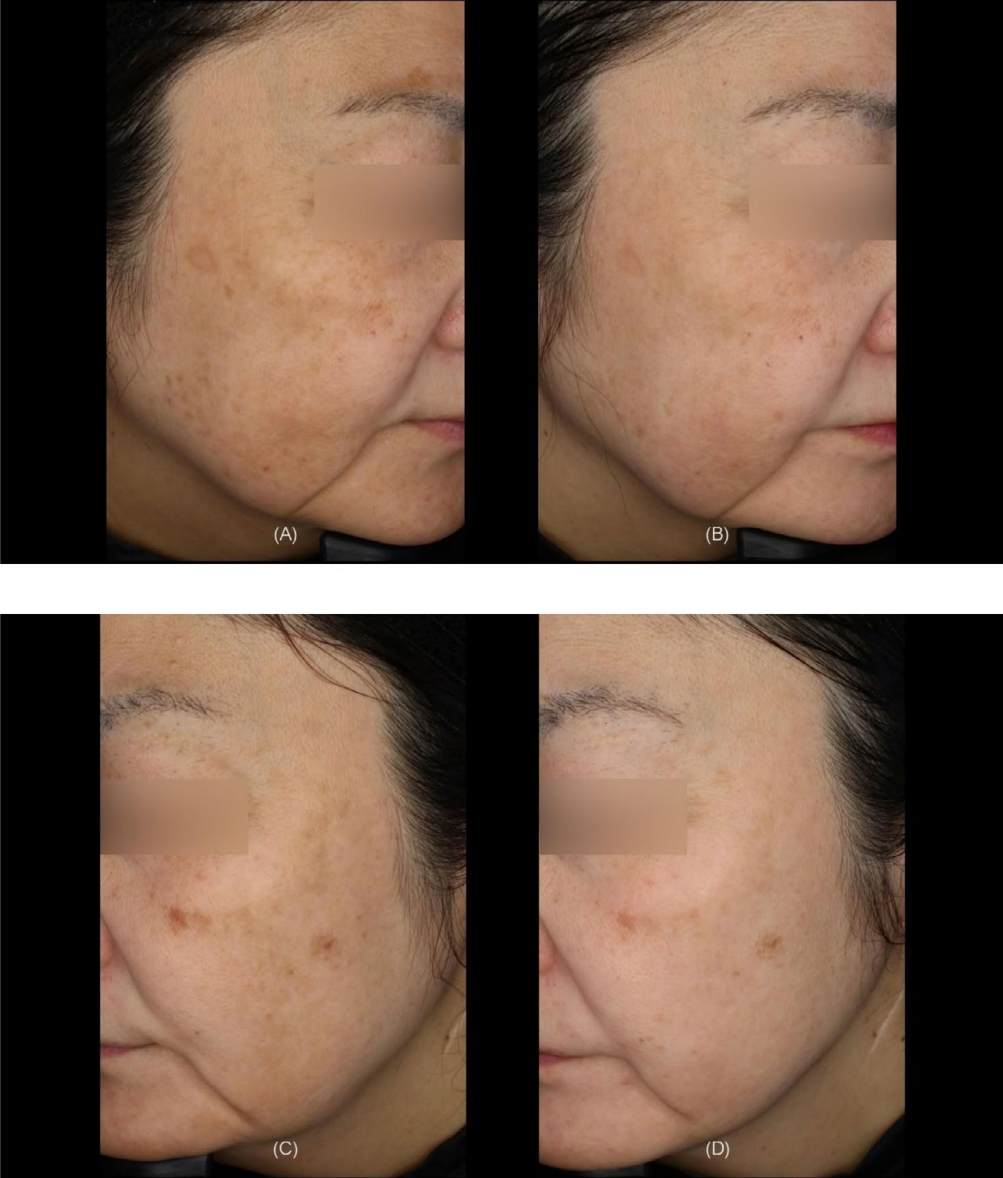
A 53‐year‐old female with Fitzpatrick Skin Type III. (A) Right side view at baseline. (B) Right side view at 6 months after the treatment. (C) Left side view at baseline. (D) Left side view at 6 months after the treatment.

The mean number of treatment shots applied per patient was 198 ± 11, with a mean total applied thermal energy of 30615.9 ± 1698.2 J per subject. Representative clinical results demonstrated marked improvements in pigmentation color, overall skin tone, and skin texture (Figures 3, 4, 5).

Pain experienced during treatment was mild to moderate, with an average rating of 2.95 ± 0.59 on a 5‐point Likert scale. Importantly, no adverse events, including burns, scarring, pigmentary alterations, or downtime, were observed or reported during the treatment or throughout the follow‐up period.

Patient satisfaction scores showed that seven subjects reported being very satisfied and thirteen reported being satisfied, while none reported being neutral, dissatisfied, or very dissatisfied.

## 4 Discussion

Previous melasma therapies primarily focus on melanocyte activity or melanin reduction through methods such as topical bleaching agents, chemical peels, and energy‐based device (EBD) treatments. However, these conventional approaches often achieve limited clinical success due to partial pigmentary improvements and frequent recurrence [7].

Recent research highlights the significant role of cellular senescence in melanocytes, characterized by the secretion of senescence‐associated secretory phenotype (SASP) factors, which contribute to chronic inflammation and impaired skin homeostasis via paracrine senescence [8]. Importantly, cellular senescence is not limited to melanocytes; senescent fibroblasts similarly induce paracrine senescence in neighboring melanocytes and keratinocytes, creating a self‐perpetuating negative feedback loop between critical cellular components within dermal and epidermal compartments, thereby exacerbating melasma pathology [9].

Previous studies investigating treatments targeting these mechanisms have demonstrated notable melasma improvements using high‐intensity focused ultrasound (HIFU). For instance, one study reported that 52% of subjects achieved more than 50% pigmentation improvement compared to controls following HIFU treatment [10], whereas another study reported improvements in 72.5% of participants [11]. These outcomes align with basic research findings indicating that HIFU enhances dermal collagen and elastin fiber synthesis in aging skin through modulation of Caveolin‐1 (Cav‐1) and suppression of p53 activity [12]. An overview of these studies and a comparison with the present study are presented in Table 2.

**TABLE 2:** Summary of comparable noninvasive energy‐based device studies using the same mechanism of action, published since 2020, that used MASI or mMASI to assess melasma.

Study/Year	Modality	Design	Population	Sessions & Follow‐up	Outcome metric	mMASI/MASI improvement	Primary findings (concise)	Safety
Vachiramon et al., 2020	HIFU (high‐intensity focused ultrasound)	Single‐blinded, randomized split‐face pilot study (HIFU+HQ vs. HQ alone)	Asians (FST III–IV); *n* = 25	3 sessions (monthly); assessments to 20 weeks	mMASI, Lightness index	15.33 ± 5.91 → 12.81 ± 6.79 (5mo, −16.4%)	Treatment side showed greater improvement; between‐side difference not statistically significant; HIFU may serve as adjuvant	Minimal AEs; no worsening of melasma
Lim, 2023	MFU‐V (superficial micro‐focused US with visualization)	Uncontrolled, pilot study	Chinese (FST III–IV); *n* = 20	2 sessions (1‐month interval); 5 months follow‐up	mMASI(per cheek)	13.2 ± 5.26 → 2.8 ± 2.24 (5 month, −78.8%)	Significant mMASI reduction; 72.5% of cheeks showed lightening; improvement maintained after 2nd session	Pain mostly mild–moderate; overall good tolerability
Present study	High‐intensity, high‐frequency parallel ultrasound beams	Uncontrolled, Prospective study	Japanese(FST II–IV); *n* = 20	Single session; 6 months follow‐up	mMASI	4.31 ± 1.37 → 1.50 ± 0.59 (6 month, −65.2%)	Mean improvement 65%; no recurrence at 6 months; texture/uniformity also improved	No AEs; pain mild(2.95 ± 0.59)

This study demonstrates the potential efficacy and safety of the Thermal‐Thread Technique, which utilizes high‐intensity, high‐frequency parallel ultrasound beams to manage melasma by specifically targeting dermal–epidermal interactions.

Contemporary immunohistochemical observations suggest that many cases previously categorized as “dermal” may, in fact, involve dermal melanocytic presence, supporting the predominance of epidermal and mixed types in clinical practice. In this study, both epidermal and mixed types were included; no difference in treatment response was observed between these categories within the limits of our sample size. Because our approach targets dermal–epidermal interactions, these findings suggest potential benefit irrespective of melasma subtype.

The Thermal‐Thread Technique used in this study specifically targets dermal remodeling and modulation by addressing senescent fibroblasts and collagen fibers, components increasingly recognized as important contributors to melasma pathology. By selectively delivering controlled thermal energy to the dermis while sparing the epidermis, this technique potentially mitigates fibroblast senescence, restores fibroblast function, and attenuates chronic inflammatory signaling. The high‐intensity, high‐frequency parallel ultrasound beam device utilized in this study delivers substantial thermal energy broadly within the dermis compared to conventional HIFU systems. Simulation data have revealed precise temperature ranges achievable at a depth of 1.5 mm, including 62.9°C at 3.0 J, 64.2°C at 3.2 J, 65.4°C at 3.4 J, 66.4°C at 3.6 J, 68.5°C at 4.0 J, 71.0°C at 4.5 J, and 73.7°C at 5.0 J. Our parameter selection was guided by these simulation data indicating that temperatures ≥ 65°C may exceed conditions favorable for biomodulatory collagen remodeling and risk coagulative effects. As our objective was dermal biomodulation rather than thermal coagulation, thus, energy settings of 3.2 J or lower were used.

Notably, our study demonstrated substantial pigmentary improvements without any adverse events or significant patient discomfort, underscoring both efficacy and safety. Moreover, clinical improvements observed extended beyond melasma reduction and included enhancements in overall skin quality. Particularly noteworthy was the observed improvement in superficial pigmentary lesions such as solar lentigines rather than structurally abnormal lesions like seborrheic keratoses. This suggests that modulation of dermal–epidermal interactions facilitated the gradual elimination of accumulated melanosomes through enhanced keratinocyte turnover. Enhanced skin quality likely contributed to more uniform light reflection, reducing the visibility of darker areas. Additionally, dermal remodeling involved increased mucin components, notably hyaluronic acid, enhancing skin barrier function, reducing transepidermal water loss, and providing increased protection against external irritants and oxidative damage. Further observed improvements in skin pore appearance, texture, and elasticity likely resulted from extracellular matrix reconstruction driven by enhanced dermal fibroblast activity.

Despite these promising findings, this study has several limitations. The sample size was relatively small, and the follow‐up period was limited to six months, constraining evaluation of long‐term efficacy and recurrence risk. Moreover, energy settings and the number of passes were not adjusted according to FST. A single‐session protocol yielded mMASI improvement rates of 59.9% for FST II, 68.5% for FST III, and 52.7% for FST IV. Although the FST IV subgroup (*n* = 2) is underpowered, the data nevertheless suggest that skin phototype may modulate response. A plausible biological basis warrants consideration: previous work indicates that neuregulin‐1 secreted by fibroblasts from darker skin increases melanocyte pigmentation and regulates proliferation in tissue‐culture and reconstructed‐skin models, which could influence clinical response. Furthermore, the cohort predominantly included mild‐to‐moderate melasma, potentially limiting generalizability to more severe presentations. Future research should incorporate larger and more diverse patient populations, multiple treatment sessions, a broader range of energy settings (from higher to lower), and extended follow‐up to comprehensively evaluate clinical efficacy, durability of results, and recurrence risks.

## 5 Conclusion

The Thermal‐Thread Technique, utilizing high‐intensity, high‐frequency parallel ultrasound beam, demonstrates substantial promise as a therapeutic modality for melasma management. By specifically targeting dermal–epidermal interactions through selective modulation of dermal fibroblast activity and mitigation of fibroblast senescence, this technique effectively addresses underlying dermal factors contributing to melasma pathogenesis. Clinical outcomes from the present study indicate not only significant pigmentary improvement but also notable enhancements in overall skin quality. Importantly, the observed therapeutic efficacy was achieved without adverse effects or significant patient discomfort. Nonetheless, further research involving larger patient cohorts, more severe melasma presentations, repeated treatment sessions, and extended follow‐up periods is warranted to comprehensively evaluate long‐term effectiveness, durability of results, and recurrence rates.

## Author Contributions

Kentaro Oku was solely responsible for all aspects of this study, including the conceptualization and study design, acquisition and interpretation of data, drafting and critically revising the manuscript, and approval of the final version. The author agrees to be accountable for all aspects of the work, ensuring that any questions related to the accuracy or integrity of the content are appropriately investigated and resolved.

## Ethics Statement

This prospective, uncontrolled clinical trial was conducted from November 2023 to November 2024 in accordance with the ethical principles of the Declaration of Helsinki. Written informed consent was obtained from all participants before enrollment. The study protocol was reviewed and approved by the Shiba Palace Clinic Institutional Review Board (Approval Number; 162120_rn‐42 485).

## Conflicts of Interest

The author declares no conflicts of interest.

## Data Availability

The data that support the findings of this study are available from the corresponding author upon reasonable request.
